# Respiratory Support of the Preterm Neonate: Lessons About Ventilation-Induced Brain Injury From Large Animal Models

**DOI:** 10.3389/fneur.2020.00862

**Published:** 2020-08-14

**Authors:** Kyra Y. Y. Chan, Suzanne L. Miller, Georg M. Schmölzer, Vanesa Stojanovska, Graeme R. Polglase

**Affiliations:** ^1^The Ritchie Centre, Hudson Institute of Medical Research, Clayton, VIC, Australia; ^2^Department of Obstetrics and Gynecology, Monash University, Clayton, VIC, Australia; ^3^Neonatal Research Unit, Centre for the Studies of Asphyxia and Resuscitation, Royal Alexandra Hospital, Edmonton, AB, Canada; ^4^Department of Pediatrics, University of Alberta, Edmonton, AB, Canada

**Keywords:** ventilation, respiratory support, ventilation-induced brain injury, neurodevelopment, preterm

## Abstract

Many preterm neonates require mechanical ventilation which increases the risk of cerebral inflammation and white matter injury in the immature brain. In this review, we discuss the links between ventilation and brain injury with a focus on the immediate period after birth, incorporating respiratory support in the delivery room and subsequent mechanical ventilation in the neonatal intensive care unit. This review collates insight from large animal models in which acute injurious ventilation and prolonged periods of ventilation have been used to create clinically relevant brain injury patterns. These models are valuable resources in investigating the pathophysiology of ventilation-induced brain injury and have important translational implications. We discuss the challenges of reconciling lung and brain maturation in commonly used large animal models. A comprehensive understanding of ventilation-induced brain injury is necessary to guide the way we care for preterm neonates, with the goal to improve their neurodevelopmental outcomes.

## Introduction

Respiratory support is a necessary life-saving intervention which has been associated with brain injury, especially in preterm neonates. Preterm birth, defined as birth prior to 37 completed weeks of gestation, is a major cause of perinatal mortality and morbidity (1, 2). Almost 1 million preterm infants who survive the neonatal period suffer adverse neurodevelopmental outcomes (1) which, in addition to an individual burden, imposes enormous financial and social costs to their families and society. Many complications associated with prematurity are due to an interruption of normal organ development that would otherwise proceed to term *in utero*. For this reason, the distinction of babies by gestational age (GA) at birth—extremely preterm (<28 weeks), very preterm (28–<32 weeks), and moderate to late preterm (32–<37 weeks)—helps to identify infant populations which are most at risk of complications related to preterm birth (3). Notably, the lungs of very and extremely preterm infants are often too immature to provide adequate respiratory function required to sustain extrauterine life.

The lower the GA of the infant at birth, the less mature the lungs are, and the higher the requirement for respiratory support. An estimated 2.4 million babies are born very and extremely preterm worldwide each year (3) and ~60–95% of these infants will require respiratory support during their neonatal period (2, 4–7). At the same time, the brains of these infants who require respiratory support are at a vulnerable stage of development and prone to injury. It is this combination of high requirements for respiratory support and the heightened vulnerability of their immature brains that increases the risk of ventilation-induced brain injury (VIBI) in extremely preterm infants.

Importantly, VIBI is likely to ensue as early as when respiratory support commences in the delivery room. Depending on GA, ~34–85% of preterm infants require intubation and positive pressure ventilation (PPV) to establish lung aeration immediately after birth (2, 8–10). These statistics exclude non-invasive forms of ventilation, meaning the total percentage of preterm infants who need respiratory support immediately after birth is substantially higher. Despite this high requirement, the limitations of equipment used in delivery suites mean that a significant proportion of babies receive inappropriate pressures or tidal volumes (V_T_) (11, 12), which can initiate pathways leading to VIBI (13). Subsequent to this, the duration of ventilatory support in the neonatal intensive care unit (NICU) is proportional to the risk of neurodevelopmental impairment and disorders (5, 14).

Respiratory support exacerbates key pathways of preterm brain injury: (1) cerebral inflammation and (2) cerebral hemodynamic instability (13, 15, 16), meaning ventilated preterm infants are in the unfortunate position of double jeopardy and are at an increased risk of brain injury. The nature of VIBI is not fully understood because it is difficult to determine clinically if brain injury is attributed solely or predominantly to ventilation. It is in this background that large animal models have played a vital role in improving our understanding of the pathogenesis of VIBI and to aid development of therapies.

In this review we will explore the issue of VIBI, how large animal models have been utilized to investigate VIBI, and the value of these models to develop much-needed therapies.

## Preterm Birth, The Requirement For Respiratory Support, And How This May Be Injurious

Prematurity is the key contributor to the need for respiratory support in newborns. The majority of extremely preterm newborns will require respiratory support due to inadequate alveolarization, insufficient surfactant production, and impaired lung liquid clearance, together with reduced respiratory drive, weak chest muscles and flexible ribs (16, 17).

Our improved understanding of respiratory transition and lung function from fetal to newborn life has led to significant advances in neonatal respiratory care, many of which aim to reduce the risk of chronic lung diseases and adverse neonatal outcomes. Despite this, a significant proportion of preterm infants still develop long-term pulmonary and neurodevelopmental morbidities due to ventilation-induced injury. Various methods of respiratory support (e.g., nasal continuous positive airway pressure, PPV via face mask or endotracheal tube) have been linked to cerebral inflammation and neuropathologies in preterm infants, including cystic periventricular leukomalacia, diffuse white matter injury and intraventricular hemorrhage (IVH) (14, 18–21). It is essential to clarify and address the effect ventilation has on the preterm infant.

### Positive Pressure Ventilation in the Delivery Room

Most infants can independently transition from a fetus to a newborn, but many preterm infants will require assistance for this physiologically challenging process. Neonatal transition involves cardiovascular adaptations and, more importantly, respiratory adaptations since the newborn is no longer supported by the placenta for oxygenation (17). Infants who cannot spontaneously breathe at birth will require PPV which is usually first delivered non-invasively via a facemask, and infants who are still unable to initiate stable respiration are intubated (22, 23). Extremely preterm infants may be electively intubated in the delivery room in some centers although it has been suggested that individualized intubation strategies after establishing respiratory failure may be better to reduce morbidities (24, 25), given that the process of intubation may itself be injurious and is associated with neurodevelopmental impairments (26).

A significant proportion of very and extremely infants require intubation in the delivery room. Despite decreasing percentages of infants requiring intubation in the delivery room over the past decades (7, 10), a staggering 31.6–77.7% of very low birth weight (VLBW) and/or extremely preterm infants continue to require this invasive intervention (2, 7, 9, 10). Early PPV in the delivery room has been associated with the development of severe IVH (20, 21). VLBW infants, mostly born extremely preterm, who received PPV in the delivery room had a nearly 3-fold increased likelihood of severe IVH (grades III and IV) than infants who did not receive PPV (20). However, it could be that the infants who require higher levels of intervention are sicker and more vulnerable to brain injury to begin with, hence it is challenging to accurately determine the extent to which advanced resuscitation is causal in the progression of brain injury in these infants.

Importantly, despite the high requirement of PPV in the delivery room, it is likely the least controlled respiratory support a neonate will ever receive, and this has proven to be inadvertently injurious to the immature brain (11, 13, 21). Current neonatal resuscitation guidelines in the delivery room rely on visual assessment of chest rise to deliver an adequate V_T_ during PPV where pressure monitoring is unavailable (23, 27, 28). Besides being subjective, the ability to observe changes in chest wall movement is reduced when a preterm infant is covered to maintain body temperature during delivery room resuscitation, stabilization, and transportation (27). It is challenging even for experienced clinicians to accurately estimate the V_T_ delivered (11, 28) and a noticeably expanded chest wall from PPV may itself be a sign of lung overdistension. Excessively high V_T_ causes volutrauma—a major cause of lung inflammation and injury (16, 29–32). Together, these factors contribute to a suboptimal ventilation situation that leads to injury of the lung and, consequently, the brain. Indeed, the use of excessive V_T_ has dire consequences on the immature brain. Preterm infants <29 weeks GA who received unintentional high V_T_ ventilation (>6 ml/kg, where median normal V_T_ is 4.2–5.8 ml/kg) in the delivery room had a nearly 4-fold higher incidence of IVH than infants who received normal V_T_ (<6 ml/kg; 51% vs. 13%) (21, 33).

Other mechanisms by which PPV leads to lung injury are barotrauma (e.g., high airway pressure), atelectrauma (e.g., repeated opening and closing of collapsed airways), and biotrauma (30–32, 34). Systemic inflammation secondary to lung injury can also initiate cerebral inflammation which is a major cause of brain injury. Inappropriate ventilation pressures and volumes can also trigger the hemodynamic pathway of injury to cause hemorrhagic brain injury (13).

### Mechanical Ventilation in the Neonatal Intensive Care Unit

Preterm infants often continue to require respiratory support after transfer to the NICU. In Australia and New Zealand, up to 95.0% of very and extremely preterm babies (<32 weeks GA) and 91.3% of moderate to late preterm infants (32–36 weeks GA) needed assisted ventilation in the NICU, with each baby receiving on average 8.8 days of assisted ventilation (2). A cohort study in South Korea reported that 38.5% of VLBW preterm infants received >7 days of mechanical ventilation (35). Importantly, the trends for long-term respiratory support in preterm infants do not seem to be decreasing (2, 36).

Prolonged periods of mechanical ventilation increases the risks of IVH (4, 22), periventricular leukomalacia or white matter injury (4, 6, 19, 35, 37), cerebral palsy (14), and attention deficit hyperactivity disorder (14) in preterm infants. In a retrospective analysis of extremely low birth weight infants, most of whom were extremely preterm, only 24% of infants who were ventilated for ≥60 days and 7% of those ventilated for ≥90 days survived without neurodevelopmental impairments (5). All infants who had been ventilated for ≥120 days and survived suffered some form of neurodevelopmental impairment (5).

Compared to the initiation of PPV in the delivery room, PPV in the NICU is much more controlled with sophisticated equipment and vigilant monitoring of ventilation parameters (38). The precise cause of VIBI in this setting has not been thoroughly investigated, with additional confounding factors such as analgesia and anesthetics (39–41), oxygenation (19), and a plethora of other NICU interventions for a range of primary and/or secondary complications that need to be considered. However, it is known that the duration of ventilation is an important determinant of neurodevelopmental morbidities (5, 19, 42).

Attempts to shift management encouraging earlier extubation or less invasive ventilation strategies have not translated to improved neurological outcomes in preterm infants (43). Furthermore, limiting the duration of mechanical ventilation to reduce complications is not always feasible with preterm infants. Therefore, it is imperative to devise treatments for unavoidable brain injury from prolonged respiratory support.

## Using Animal Models to Investigate Ventilation-Induced Brain Injury

Clinical observations discussed above underpin the need to understand mechanisms through which respiratory support causes brain injury, to allow focused clinical strategies or new therapies aimed at improving outcomes. However, such investigations are not necessarily achievable in preterm infants since respiratory support cannot be studied in isolation. Herein lies the value of using animals for comprehensive characterization of VIBI through imaging, physiological, immunohistochemical, and molecular techniques. Animals can also be used to model various conditions such as growth restriction and chorioamnionitis to more closely interrogate VIBI under conditions of compromised pregnancies.

**Figure 1:**
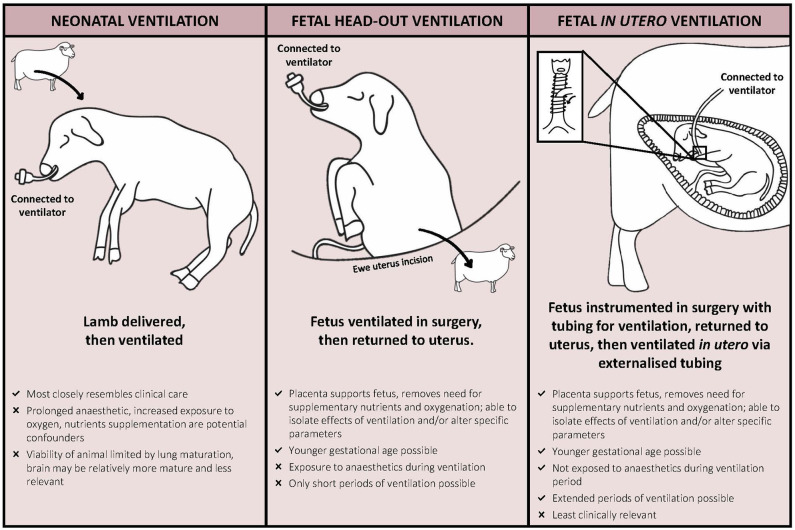
Experimental models of ventilation-induced injury in sheep outlining the major advantages and disadvantages of each model.

Studies using large animals, most often sheep, to model ventilation-induced injury can be categorized by experimental technique (fetal [head-out or *in utero* ventilation] or neonatal ventilation) and period/duration of ventilation (acute or chronic) (Figure 1). These different experimental techniques enable replication of specific scenarios of preterm respiratory support, including the initial resuscitation in the delivery room and prolonged care in the NICU. However, with all models, an understanding of the strengths and limitations is essential for appropriate interpretation of the findings and potential replication in clinical trials.

### Balance Between Lung and Brain Development in Large Animal Models

An inevitable limitation to using large animals to model neonatal conditions is the difference in developmental milestones of major organ systems and physiology compared to humans. The animal's age cannot be chosen based on gestation duration alone since an animal at 0.65 gestation is not necessarily developmentally equivalent to a human at 0.65 gestation. Instead, the crucial factor is the stage of development of the organ of interest. This proves challenging for models of VIBI as the developmental milestones of both the brain and lungs must be considered. Detailed comparisons of species-specific lung development and anatomical features have previously been compiled (44–46) and comparisons for brain development are summarized in Table 1. This presents a conundrum: how do we balance desired stage of brain development with lung maturation?

**Table 1:** Comparative gestational ages for key brain development processes in the human, baboon, and sheep.

**Developmental process**	**Human (term 40 weeks)**	**Baboon (term 185 days)**	**Sheep (term 148 days)**
**Weight**
Growth spurt	26–28 wk	125–140 d	85–100 d
**Cortical folding**
Primary	*ev* 26–28 wk	*ev* 125 d	*ev* 71–89 d
Secondary	*ev* 32–34 wk	*ev* 140 d	n.d.
Tertiary	*ev* 40–44wk	*ev* 160 d	n.d.
Six distinct cortical layers	ev 28 wk	*ev* 125 d	*ev* 89 d
**Neurogenesis and Gliogenesis**
Main neuronal multiplication	10–15 wk	n.d.	40–80 d
Main glial multiplication	36–40 wk	n.d.	95–130 d
**Myelination**
Periventricular white matter (preOL predominant)	23–28 wk	n.d.	93–99 d
Internal capsule	*ev* 32 wk	*ev* 125 d	*ev* 78–96 d
Superior temporal gyrus	Mature at 48 wk	Moderate at 160 d	n.d.
Cerebellum	*ev* 28 wk	*ev* 125 d	*ev* 80 d

*d, days; ev. evident at; n.d., not determined; OL, oligodendrocytes; wk. weeks*.

*Note that there is often heterogeneity in development not just between different regions in the brain, but within each region. Compiled from refs human and cross-species comparisons (19, 47–50), baboon (51–53), sheep (54–61)*.

Non-human primate studies have used baboons (*Papio papio*; *Papio cynocephalus*) delivered at 125 days (term is 185 days; 0.68 gestation) which have similar lung development to an infant born at 26 weeks preterm age (44). At this stage, brain development is comparable to that of a 26–28 week-old extremely preterm human infant (51). While these developmental stages are congruent, there are significant technical challenges as well as practical, financial and ethical concerns associated with the use of non-human primates (62). Thus, even though they are the closest animal models to humans and offer vital insights to developmental studies (62), non-human primate models of VIBI are relatively less commonly pursued.

Piglets (*Sus scrofa*) have been proposed to be suitable for studying neurodevelopment and cerebral consequences of early life insults (63). A piglet at 91–94 days gestation (term is 115 days; 0.8 gestation) is physiologically similar to a 23–25 week extremely preterm infant in terms of lung development and the requirement for respiratory support for survival (64). Neurodevelopment of the piglet at this GA is slightly more mature, comparable instead to a moderate to late preterm human infant (63, 65). Besides this developmental mismatch, there are significant challenges in performing fetal surgery and chronic instrumentation in pigs due to relatively large litters and the large size of the sow. Hence, piglets have not been widely used in VIBI studies that require these techniques. The suitability of piglets in postnatal ventilation studies for VIBI has not been extensively explored.

Similarly, brain maturation in sheep (*Ovies aries)* advances more rapidly in late gestation than development of the lungs, relative to humans. Previous studies in preterm lambs that have investigated VIBI were performed at the earliest GA at which the lambs were viable with respiratory support [125 days where term is 148 days; 0.85 gestation; structural lung development comparable to a 26–28 week human infant (46, 66)]. However, a limitation is that the fetal sheep brain development at 125 days gestation is comparable to a late preterm or term human fetus on the basis of white matter maturation (60, 61). Studies using lambs at this gestation have investigated the effect of respiratory support for up to 4 weeks on chronic lung injury (67), but have only looked at VIBI up to 24 h of ventilation (68).

### Modeling of Acute VIBI

Animal studies to date have focused on VIBI downstream of lung injury resulting from volutrauma in the delivery room. The rationale behind these studies is that clinical findings have shown that variable V_T_ during delivery room resuscitation can be outside recommended limits (11, 12, 69).

Observed V_T_ during mask ventilation can range from 0 to 31 ml/kg (11, 12, 69) and V_T_ during endotracheal tube ventilation has been reported to be 3.9–9.6 ml/kg (12). The upper ranges of these V_T_ are higher than the recommended 4–8 ml/kg for very and extremely preterm infants (11). This is critical as we have known for decades that as few as six manual inflations of high V_T_ (35–40 ml/kg) are enough to induce injury in immature, surfactant-deficient lungs of preterm lambs (70). Sheep studies that have investigated acute VIBI have similarly found that brief periods of high V_T_ ventilation resulted in detectable brain injury as early as 90 min after ventilation onset (13, 15, 16, 71–73). Importantly, even if recommended V_T_ is delivered, the act of respiratory support in itself can activate an immune response in immature respiratory units (30, 31, 34).

To isolate this initial period of injurious respiratory support, akin to poorly regulated V_T_ in the delivery room, sheep studies have employed an acute high V_T_ ventilation strategy: 15 min injurious ventilation with stepwise increments of V_T_ to achieve a high target V_T_ of 10–15 ml/kg, which is 2–3 times the normal V_T_ of lambs for that GA (125 days of term 148 days; ~5–7 ml/kg) (13, 15, 16, 71–73). Thereafter, lambs are sustained on appropriate respiratory support (neonatal model) or returned to the uterus (head-out model) to allow for the inflammation and injury pathways to manifest into gross lung/brain injury. These studies have provided valuable information on the pathology and mechanisms of acute VIBI (discussed in section Understanding VIBI From Injurious Respiratory Support in the Delivery Room).

### Chronic Models of VIBI

The majority of animal models used to model VIBI are acute, focusing on the initial hours after birth. This is mainly due to inherent problems with maintaining animals for long periods of time. In particular, the problem with maintaining respiratory support for long periods of time in newborn animal models is the inability to control for specific factors, due to the need to introduce increasing levels of neonatal intensive care—akin to that of looking after a chronically ventilated preterm infant. To get around this problem, animal models have utilized respiratory support via a head-out approach or entirely *in utero* (Figure 1). Using these techniques, the intact placental circulation manages nutrition and gas exchange of the fetus, allowing subtle mechanisms of respiratory support to be examined. In the head-out approach, the fetal head and chest are exteriorized, the fetus is intubated and ventilation with various strategies altering delivered volume, pressures, respiratory frequencies, or oxygen content, and then returned to the uterus (74–78). *In utero* ventilation (IUV) studies require the fetus to be exteriorized and instrumented with ventilation tubes and equipment required for monitoring prior to being returned to the uterus. After a recovery period for the ewe and fetus, the fetus is ventilated via the externalized ventilation tubes for various times, although to date the longest has been 12 h (79–81). IUV has been used in fetal sheep to study cardiopulmonary physiology (82, 83), lung mechanics (84), and ventilation-induced lung injury (79, 81, 85). While cerebral physiological responses to IUV have been investigated previously (86), histopathology of brain injury after IUV has not been reported.

It is obvious that these *in utero* models are not designed with the intention to replicate clinical situations given that prolonged neonatal studies are more reflective of current clinical care. Instead, they provide the opportunity to manipulate specific ventilatory parameters in isolation so that we can better understand the contribution of a sole variable to lung and brain injury. Importantly, the IUV model allows ventilation of a fetus at a younger gestation than would be viable postnatally. This is advantageous, especially in ovine models, as the stage of brain development will be more comparable to that of extremely preterm infants.

## Understanding VIBI From Injurious Respiratory Support in the Delivery Room

### Animal Studies That Investigate Pathology of VIBI

Studies in preterm lambs have characterized acute white matter changes following 15 min of injurious high V_T_ ventilation (13, 15, 16, 71–73, 87, 88). High V_T_ ventilation causes a robust pulmonary inflammatory response which increases systemic and cerebral inflammation, characterized by elevated IL-6 and IL-8 messenger ribonucleic acid (mRNA) levels in the periventricular and subcortical white matter of the brain in ventilated preterm lambs (15, 73, 87). Increased microglial activation and aggregation, and a higher incidence of vascular protein extravasation (indicative of a compromised blood-brain barrier) and cerebral hemorrhage in the same regions were also observed (15, 73, 88). Injurious ventilation did not alter expression of myelin basic protein (MBP; oligodendrocyte marker) in the internal capsule or neuronal nuclei (NeuN; neuron marker) in the thalamus (89) and did not increase inflammation or injury in gray matter (90).

Importantly, pathology resultant from injurious ventilation can be visualized using non-invasive imaging such as magnetic resonance imaging (MRI) (72, 77, 91) and correlated with histopathology (89). Magnetic resonance spectroscopy (MRS) detected acute changes in brain metabolite peak-area ratios (Lactate/Creatine and Lactate/Choline) in preterm lambs that received high V_T_ although macroscopic injury was absent in structural MR images (T1, T2) (72). Alterations in MRS-detected metabolite levels relate to neuronal damage and potentially predict subsequent neurodevelopmental impairments (92, 93). Notably, these MRS changes were observed within 90 min of ventilation onset (72). Recent findings suggest that MRS-detectable changes persist 24 h after injurious ventilation (77). Diffusion tensor imaging (DTI) perhaps offers the most sensitive measures of early brain injury. DTI detected decreased diffusivity measures in the frontal white matter (axial, radial, and mean) and internal capsule (axial) in preterm lambs 24 h after injurious ventilation (77). These parameters have been suggested to correlate with myelination deficits (77).

### Mechanistic Insight From Animal Studies

Several explanations have been put forward to link ventilation and brain injury. Studies in ventilated preterm lambs have identified two major pathways of acute VIBI: cerebral inflammation and hemodynamic instability (13, 15, 16, 77). Both pathways are proposed to be downstream effects of the pulmonary consequences following ventilation (13, 15, 16). Incidentally, these key VIBI pathways mirror those of preterm brain injury—suggesting compounded risk of injury in preterm infants. These mechanisms have been reviewed previously (13).

Briefly, the inflammatory pathway of VIBI involves upregulation of pro-inflammatory cytokines (e.g., IL-6, IL-8) and activation of microglia and astrocytes within the developing white matter of the brain (94). Injurious ventilation initiates a profound pulmonary inflammatory response caused by volutrauma, barotrauma, atelectrauma, and/or biotrauma (30–32, 34). This inflammatory cascade is associated with systemic inflammation and subsequent localized inflammation and injury in the white matter involving glia cells (13, 15, 16). Activated microglia and astrocytes are thought to mediate the destruction of cells in the oligodendrocyte lineage, contributing to hypomyelination and diffuse white matter injury that can underlie long-term neurological sequelae such as cerebral palsy (94, 95).

The hemodynamic pathway of injury refers to significant alterations, caused by PPV and atypical to hemodynamic changes during the transition at birth, to pulmonary blood flow and consequently cardiac output and cerebral blood flow (CBF) (15, 16). During PPV, applying a high pressure into the airways decreases pulmonary capillary transmural pressure, causing compression of intra-alveolar capillaries, hence increasing capillary resistance and decreasing pulmonary blood flow (13, 16). This reduces pulmonary venous return, left ventricular output, and accordingly alters CBF (13, 15, 16). Arterial blood pressure variability within a physiological range is not usually a problem because it is compensated by pressure-flow autoregulation to sustain a stable CBF. This involves constriction and dilation of arteries to alter cerebral vasculature resistance in response to changing perfusion pressures (96). The autoregulatory plateau, bounded by lower and upper limits of arterial pressure, has been postulated to be narrower in preterm infants with decreasing GA (96–98). Moreover, it has been suggested that preterm delivery or treatments reflective of clinical care of the preterm infant, including mechanical ventilation, affects cerebral autoregulation (99). Prolonged CBF fluctuations for more than 10 to 20 s has been defined as cerebral hemodynamic instability (100). The initiation of ventilation in preterm lambs caused CBF instability in the initial 15 min, even when a gentle strategy was used (71); the variability in CBF amplified when an injurious high V_T_ strategy was used (15). Clinically, 91% of babies with respiratory distress syndrome who had fluctuating CBF after 12 h of life subsequently had an IVH (101), highlighting the critical importance of preventing fluctuations in hemodynamics immediately after birth.

The relative contribution of each pathway toward the progression of white matter injury discussed in section Animal Studies That Investigate Pathology of VIBI is unknown although a recent study suggests that the hemodynamic pathway has an additive effect on the inflammatory pathway on injury progression, but the inflammatory pathway seems to dominate (77).

## Understanding VIBI From Ventilation in the Neonatal Intensive Care Unit

Ventilation studies in preterm baboons and lambs suggest that the brain injury underlying neurodevelopmental impairments in chronically ventilated preterm infants involves subtle diffuse white and gray matter lesions, often without intraventricular or germinal matrix hemorrhage and overt lesions or infarcts (19, 51, 102–104). This indicates a potentially distinct mechanism of injury to acute VIBI sustained in delivery room settings.

Preterm baboons have been used extensively to study the impact of prolonged mechanical ventilation (2–4 weeks) on the lungs (105–107) and, more recently, the brain (103, 107, 108). In these studies, preterm baboons (125 days of term 185 days; 0.68 gestation) are cared for with similar interventions to that of preterm infants in the NICU, including mechanical ventilation using a gentle strategy to maintain V_T_ at 4–6 ml/kg with adequate chest motion (51, 106). While not investigating injury from ventilation *per se*, the brain injury observed in these animals is not from any direct insult or influenced by potentiating conditions associated with preterm birth or an adverse uterine environment. The subtle neuropathologies from preterm birth and subsequent intensive care alone closely resemble what is observed clinically (51, 102, 103, 109). After 14 days of ventilator support, preterm baboon brains had delayed gyrification (102, 104), reduced brain weight (102–104), reduced white and gray matter volumes (103, 104), increased white and gray matter injury (51), increased astrogliosis in the forebrain (103), increased ramified microglia (103), and a reduction of oligodendrocytes (103, 104) compared to gestation-matched controls. These histopathological indices correlated with microstructural and macrostructural changes detected by *ex vivo* MRI (109).

Additionally, the effects of shorter durations of controlled NICU respiratory support have been investigated in sheep. Preterm lambs (125 days of term 148 days; 0.85 gestation) ventilated with a non-injurious strategy (V_T_ at 5–7 ml/kg) had increased IL-8 and connective tissue growth factor (CTGF) mRNA levels and decreased vascular occludin protein density in the white matter after 2 h (110). When the length of ventilation was extended to 24 h, ventilated lambs had increased astrogliosis within cortical gray matter but otherwise no apparent neuropathology or changes in glial cell populations compared to unventilated control lambs (68).

Both the preterm baboon and lamb models discussed are neonatal ventilation models. However, as mentioned above, a disadvantage of the neonatal ventilation model is the intensive care requirements of maintaining a preterm animal for significant periods of time, making them more akin to human studies where individual parameters cannot be teased apart unless large numbers of animals are used, which is financially unviable. This is where the IUV model may be advantageous if used for extended periods beyond 24 h.

## Influence of the Antenatal Environment on Respiratory Support and VIBI

Work explored in the previous sections have studied the pathology and mechanisms of VIBI in preterm but otherwise healthy animals. The ability to isolate effects of respiratory support with minimal confounding factors is vital and these findings provide a foundation to explore therapeutic options to minimize VIBI which will be discussed in section Bench to Bedside of this review. However, it is important to consider that the clinical situation is much more complex—many preterm infants will have been exposed to adverse uterine environments which may increase their risk of VIBI.

### Adverse Antenatal Conditions Alter Responses to Postnatal Respiratory Support

Adverse antenatal conditions such as fetal growth restriction (FGR) and intrauterine inflammation have independently been associated with adverse neurodevelopmental outcomes in preterm infants (111, 112). Further, these infants often require respiratory support after birth, increasing the risk of brain injury. Yet, there is a paucity of information on how these antenatal conditions alter the response these infants have to ventilation and if this contributes to VIBI.

#### Fetal Growth Restriction and VIBI

FGR is a condition where the fetus fails to reach its projected growth potential, often due to placenta insufficiency (112). FGR fetuses are sometimes delivered preterm to prevent deterioration in an adverse *in utero* environment (112), thus many will require respiratory support due to prematurity. FGR fetuses have altered cardiovascular and vascular function, most notably the characteristic “brain-sparing” phenomenon by redirecting blood flow and oxygen delivery to important organs including the heart, adrenals, and brain. These adaptations persist to early postnatal life and may affect how a growth-restricted infant responds to ventilation. Preterm growth-restricted lambs ventilated with a gentle non-injurious strategy for 24 h had disrupted interaction of astrocyte end-feet with cerebral blood vessels, increased microgliosis, and increased oxidative stress compared to their unventilated counterparts and to ventilated preterm appropriately-grown lambs (68). Notably, differences between growth-restricted and appropriately grown lambs were evident after 2 h of ventilation (110). This suggests that growth restricted infants may be at increased risk of VIBI, perhaps in part due to differences in the neurovascular unit and blood-brain barrier properties (68, 110).

#### Intrauterine Inflammation and VIBI

Intrauterine inflammation, which most commonly presents as chorioamnionitis, is a major cause of preterm birth (113). Antenatal inflammation alters the vulnerability and response of the immature brain to ventilation (71, 91, 114). Lipopolysaccharide(LPS)-mediated inflammation *in utero* amplified cerebral hemodynamic instability during the initiation of ventilation in preterm lambs (71). Compared to saline controls, these lambs that had been exposed to LPS 2 or 4 days before preterm delivery had increased inflammation, vascular extravasation, and microhemorrhages in cerebral white matter regions after ventilation (71). Further, injurious ventilation increased the number of apoptotic cells (TUNEL^+^ cells) in the subcortical white matter of LPS-exposed lambs, compared to their unventilated counterparts (114). Injurious ventilation had no obvious acute detrimental effects on white matter (89) and gray matter (90) compared to injuriously ventilated healthy preterm lambs and to LPS-exposed lambs that received gentle ventilation. Brain macro- and microstructure as assessed by MRI and DTI were similarly not different (89, 91). However, a novel DTI color map threshold technique detected lower diffusivity indices in white matter regions of the brain, indicative of subtle brain injury in the ventilated lambs that were exposed to inflammation prior to delivery (91). Importantly, using a non-injurious ventilation strategy did not mitigate VIBI in the LPS-exposed lambs (114). Clinically, histologic chorioamnionitis is associated with a longer cumulative duration of mechanical ventilation in VLBW infants (35), thereby increasing the risk of VIBI. However, the combination of chorioamnionitis and prolonged ventilation has not been investigated in large animals and the potential cerebral effects are unknown.

### Cerebral Effects of Antenatal Medical Interventions

Corticosteroid administration is a common antecedent to preterm birth, where antenatal glucocorticoids (betamethasone and dexamethasone) are given to accelerate fetal lung maturation before preterm labor (115). Clinically, antenatal glucocorticoid administration is suggested to reduce the incidence and severity of IVH (115) and does not affect subsequent development of subsequent childhood mental and behavioral disorders in preterm infants (116). However, information on its interaction with respiratory support is scant. A recent study found that antenatal betamethasone improved cerebral hemodynamic instability in preterm lambs that received 15 min of high V_T_ injurious ventilation followed by 75 min non-injurious ventilation (88). However, there was an increase in the percentage of amoeboid microglia in the periventricular white matter, the number of vessel profiles with protein extravasation in the subcortical white matter, and malondialdehyde levels in cerebrospinal fluid, suggesting increased inflammation and oxidative stress in betamethasone-treated animals than their saline-treated counterparts ventilated with the same protocol (88). This potential increased risk of VIBI following antenatal betamethasone administration may lie in the increased lung compliance and hence susceptibility of the lungs to volutrauma rather than a direct cerebral effect (88).

It is crucial to consider that antenatal glucocorticoid administration may have additional interactions with the conditions mentioned above; for example, growth restricted fetuses have different hemodynamic responses to antenatal glucocorticoids compared to appropriately grown fetuses (117). Maternal betamethasone administration increased fetal cardiac output and blood flow to major organs whereas cardiac output was decreased and blood flow to major organs remained unchanged in control fetuses (118). Furthermore, there were transient decreases in carotid blood flow, an index for CBF, in both control and FGR fetuses. While CBF of control fetuses were stable after returning to baseline levels, FGR fetuses displayed a persistent rebound increase in carotid blood flow from 10 h after treatment (119). Whether these altered responses are beneficial or harmful in the context of VIBI needs to be ascertained. Indeed, little is known about the combined effects of adverse antenatal conditions, antenatal glucocorticoid administration, and postnatal respiratory support on brain injury in the preterm infant. Large animal models of VIBI may provide a means to address this.

## Bench to Bedside

Establishing reliable animal models with reproducible neuropathology that is reflective of injury seen clinically expedites efforts to test novel potential interventions and/or therapeutic candidates. Potential treatments for VIBI and their mechanisms of actions have recently been discussed in detail by Barton et al. (120) and this remains an active area of research.

In the delivery room setting, physiological-based cord clamping (PBCC) can stabilize pulmonary, systemic, and cerebral circulation in preterm (121, 122) and near-term lambs (123), essentially mitigating the hemodynamic pathway of injury, but it is unlikely to prevent VIBI resultant from the inflammatory pathway. PBCC refers to delaying umbilical cord clamping until respiration has been initiated and established in the newborn or providing respiratory support prior to umbilical cord clamping (121–123). Thus, a therapy that targets both pathways of VIBI, with a focus on modulating inflammation, is required. To date, animal experiments have investigated short-term effects of erythropoietin (EPO) and human amnion epithelial cells (hAECs) as prophylactic postnatal treatments for VIBI resultant from acute volutrauma (73, 87, 124). These treatments have proposed mechanisms of action that make them ideal candidates for neuroprotection. EPO has anti-inflammatory, anti-apoptotic, and neurotrophic properties while hAECs are anti-inflammatory and reparative (120).

When administered to preterm lambs that received 15 min of injurious high V_T_ ventilation, single early low doses of 300 IU/kg and 1,000 IU/kg human recombinant EPO did not reduce or exacerbate lung and brain injury (124, 125), suggesting that EPO doses presently used in clinical trials appear to be safe for preterm infants receiving respiratory support. However, they appear to not be efficacious as a therapy for VIBI given the lack of therapeutic potential observed. High doses of EPO of 3,000 IU/kg and 5,000 IU/kg increased cerebrospinal fluid EPO levels to “neuroprotective levels” [>100 mU/ml (126)] within 2 h of administration (73, 124). These high doses, respectively, had a protective effect on blood-brain barrier integrity (124) and differential regional effects on white matter (73) despite both doses amplifying lung inflammation and injury (125, 127). Together, these data highlight a complex dose response with distinct effects on the lungs and brain, indicating that further investigation is required to elucidate the efficacy of EPO in the context of a preterm infant requiring respiratory support.

In a similar study, preterm lambs that received high V_T_ ventilation were administered an intratracheal infusion of 9 × 10^7^ hAECs before ventilation onset and an additional intravenous dose of 9 × 10^7^ hAECs within 5 min of delivery (total 1.8 × 10^8^ hAECs) (87, 128). The cells were able to enter the brain within 2 h of administration, as detected by fluorescent cell labeling in the frontal and parietal periventricular and subcortical white matter of the brain (87). Cell administration reduced microgliosis and vascular protein extravasation (87), potentially as a downstream effect of reduced pulmonary inflammation (128). However, hAECs did not stabilize hemodynamic transition or modulate systemic inflammation within the brief period of the experiment, and conversely they induced an increase in pro-inflammatory cytokine mRNA levels within the brain (87, 128). Further long-term effects of hAECs on acute VIBI have not been investigated to accurately determine the interaction of hAECs and ventilation on the preterm brain.

Chronic ventilation studies in animals have so far focused on treatments to reduce lung rather than brain injury. Sheep fetuses ventilated *in utero* for 12 h and administered intratracheal infusion of 3 × 10^7^ hAECs and an intravenous dose of 3 × 10^7^ hAECs at 3 h and 6 h after ventilation onset (total 1.2 × 10^8^ hAECs) demonstrated a reduction in ventilation-induced lung injury (81), and as such may have the potential to reduce VIBI but this remains a speculation.

**Figure 2:**
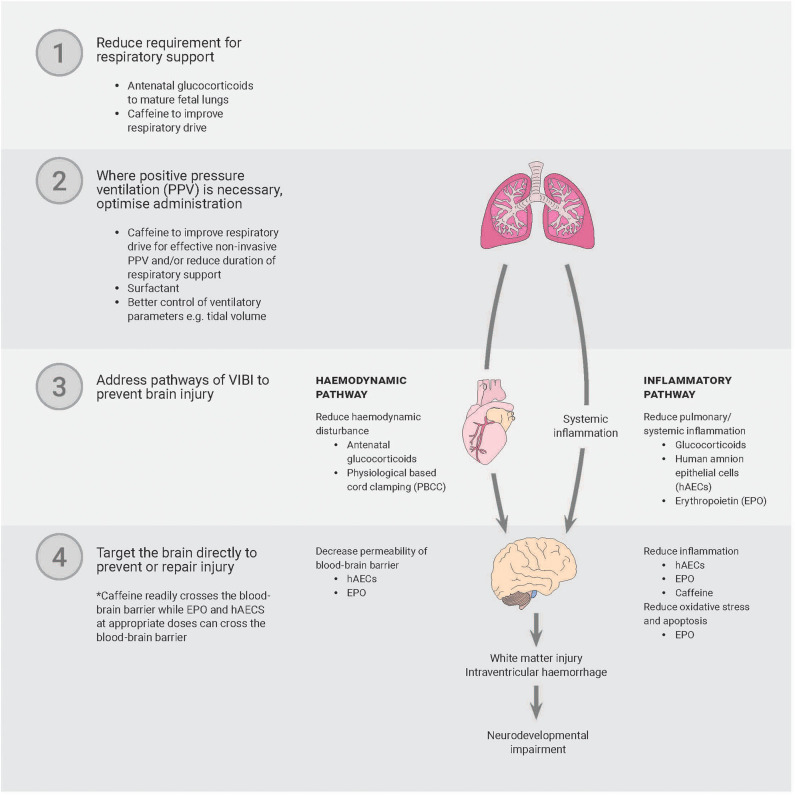
A multipronged approach is likely necessary to prevent or reduce ventilation-induced brain injury. Only strategies discussed in-text have been included in the diagram. PPV, positive pressure ventilation; PBCC, physiological-based cord clamping; hAECs, human amnion epithelial cells; EPO, erythropoietin.

While the above strategies have some promise, it is unlikely that a single strategy will prevent or reduce VIBI. The key might lie in a multipronged approach that involves reducing the requirement for or duration of respiratory support, optimizing how PPV is administered to avoid adverse effects, and reducing the sequelae of unpreventable adverse effects of PPV (Figure 2).

In this regard, researchers in The Netherlands have investigated the use of caffeine in the delivery room, showing improved respiratory efforts of preterm infants (24–30 weeks GA), potentially reducing the need for invasive respiratory support in this setting (129). Further, spontaneous respiratory drive is a determinant of effective use of gentle non-invasive respiratory support (130). However, caffeine administration to mechanically ventilated preterm infants (23–30 weeks GA) in the first 5 days of life did not encourage early extubation or decrease ventilation duration in the NICU (130, 131). The trial was terminated due to safety concerns, making it difficult to interpret results and secondary outcome findings of morbidities including BPD and IVH (130, 131). In contrast, caffeine had neuroprotective effects in very preterm infants when assessed at 18 months' corrected age (132, 133), in part attributed to earlier discontinuation of PPV and decreased rates of bronchopulmonary dysplasia (134, 135). The treatment benefits of caffeine administered in the first 10 days of life on neurobehavioral and functional cognitive outcomes were less pronounced at 5- and 11-years follow-up, with only slight but statistically significant improvements to motor outcomes observed (136–138). Earlier administration of caffeine within the first 2 days of life has been associated with improved neurodevelopmental outcomes at 18 to 24 months' corrected age compared to late administration (133) but whether these benefits persist have not been reported. Certainly, these contradictory findings highlight the need to better understand the interaction of caffeine, respiratory support, and neurodevelopmental outcomes. Independently, caffeine has been postulated to have neuroprotective properties by reducing inflammation, reducing periventricular white matter injury, and stabilizing hemodynamics in preterm infants (133).

Correspondingly, protective ventilation strategies have reduced brain inflammation and vascular protein extravasation but do not completely mitigate injury in preterm lambs (15, 16, 72, 114). Together, these indicate that our current efforts to minimize the need for respiratory support and, where respiratory support is necessary, improve the way PPV is administered are inadequate to prevent VIBI. Large animal models will likely play a key role in studies focusing on stimulating respiratory function at birth and optimizing the delivery of non-invasive respiratory support to minimize lung and brain inflammation and injury. Additionally, there is an apparent need to devise treatments and large animal models of VIBI provide a means to address this.

## Summary

This review highlights the necessity of large animal models when investigating the relationship between invasive respiratory support, the lungs, and the brain in the preterm infant. These models provide a powerful research tool; a combination of physiological, histological, molecular and imaging techniques provides an integrated picture of the interactions between respiratory support and the immature brain which is difficult to obtain in a clinical setting.

Recognizing the consequences of respiratory support on the immature brain will encourage development of effective therapies to prevent or treat VIBI in otherwise healthy preterm infants. VIBI should also be considered when investigating treatments for other conditions such as FGR, chorioamnionitis, and hypoxic injury where the compromised infant will often receive respiratory support.

## Author Contributions

KC contributed to the conception of the review and created the table. KC and GS conceptualized and created the figures. KC, SM, GS, VS, and GP contributed to structuring, drafting, revising, and the final approval of the version to be published. All authors contributed to the article and approved the submitted version.

## Conflict of Interest

The authors declare that the research was conducted in the absence of any commercial or financial relationships that could be construed as a potential conflict of interest.
